# Early cognitive comorbidities before disease onset: A common symptom towards prevention of related brain diseases?

**DOI:** 10.1016/j.heliyon.2022.e12259

**Published:** 2022-12-14

**Authors:** Laetitia Chauvière

**Affiliations:** Radboudumc, Department of Cognitive Neuroscience, Trigon Building / Route 205, Kapittelweg 29, Nijmegen 6525, EN, the Netherlands

**Keywords:** Cognitive deficits, Prevention, Prediction, Related brain diseases, Brain network abnormalities, Risk factors, Temporal lobe epilepsy, Schizophrenia, Alzheimer’s disease, Parkinson’s disease, Autism, At-risk subjects

## Abstract

Brain diseases are very heterogeneous; however they also display multiple common risk factors and comorbidities. With a paucity of disease-modifying therapies, prevention became a health priority. Towards prevention, one strategy is to focus on similar symptoms of brain diseases occurring before disease onset. Cognitive deficits are a promising candidate as they occur across brain diseases before disease onset. Based on recent research, this review highlights the similarity of brain diseases and discusses how early cognitive deficits can be exploited to tackle disease prevention. After briefly introducing common risk factors, I review common comorbidities across brain diseases, with a focus on cognitive deficits before disease onset, reporting both experimental and clinical findings. Next, I describe network abnormalities associated with early cognitive deficits and discuss how these abnormalities can be targeted to prevent disease onset. A scenario on brain disease etiology with the idea that early cognitive deficits may constitute a common symptom of brain diseases is proposed.

## 1 Introduction

Brain diseases are a societal, economic, and health challenge, with ∼1 in 3 European citizens' lives being affected each year, and cost Europe an estimated at around €798 billion [1, 2]. There is a paucity of disease-modifying therapies for brain diseases and so preventive measures have become a health priority [3, 4, 5, 6, 7, 8, 9, 10, 11, 12, 13, 14, 15, 16, 17, 18, 19, 20, 21, 22]. Brain diseases are clinically heterogeneous, but i) epidemiology shows high rates of co-occurrence [23, 24]; ii) there are similar genetic risk factors such as copy number variants [25, 26]; and iii) cognitive and mood comorbidities are common [27]. Commonly cognitive or affective symptoms precede the core disease symptoms [28, 29, 30]. A hypothesis is that these cognitive comorbidities could constitute a broad biomarker of brain diseases and reveal common endophenotypes.

In the current review, considering mostly recent publications, I will address common risk factors and comorbidities across related brain diseases with a key focus on cognitive comorbidities before disease onset. Network dynamics abnormalities underlying early cognitive comorbidities across brain diseases will also be described. I will focus on neurological and psychiatric diseases, especially on epilepsy (particularly temporal lobe epilepsy), schizophrenia, Alzheimer’s and Parkinson’s diseases, with the idea that brain diseases may share a common cause which can express itself through the emergence of early cognitive deficits and progress through the occurrence and interplay of risk factors.

## 2 Common risk factors among brain diseases

Brain diseases possess a lot of common risk factors, including genetic (e.g., risk genes, copy number variants), environmental (e.g. stress, urban rearing), and neurodevelopmental (e.g., obstetrical complications, cortical malformation) [31, 32]. These risk factors often interact. It is for example currently well-admitted that schizophrenia and bipolar disorder, both highly heritable and severe psychiatric disorders, result from the complex interaction between genetic and environmental risk factors [33] (cf. Table 1).

**Table 1:** Common risk factors among related brain diseases. This table is not exhaustive. *Abbreviations*: SCZ: schizophrenia; BD: Bipolar disorder; AD: Alzheimer’s disease; ADS: Autism spectrum disorders PFC: Prefrontal cortex; VMPFC: ventromedial PFC; HPC: hippocampus; DMN: default mode network; E-I: Excitatory-Inhibitory; IQ: Intellectual Quotient.

Categoriesof risk factors	Risk factors	Brain diseases	Main findings References
*Genetic and genomic*	- shared genetic mutations - copy number variations - common risk variants	Psychiatric disorders	Overlap exists among psychiatric disorders with common genetic risk factors [34]
	- susceptibility genes (108 identified loci)	SCZ, AD, BD	Numerous susceptibility genes have been characterized - in SCZ - in AD - in BD In SCZ, various risk genes are associated with deficits in synaptic plasticity, oscillatory activity and connectivity [35, 36, 37]
	- epigenetic factors (or the complex interaction between genetic and environmental factors that influences gene expression)	SCZ	While each individual locus confers only a very small risk of developing the disease, the conversion to psychosis is rather characterized by epigenetic factors [38]
	- specific DNA methylation modifications	SCZ	In prodromal patients for SCZ, conversion to psychotic episodes is associated with specific DNA methylation modifications, in comparison with non-converters (longitudinal study) [39]
	- allied phenotypes	SCZ, BD	Proband and family studies for both early-onset SCZ and pediatric bipolar disorders, with SCZ and BD sharing common genetic risk factors at several loci [40]
	- copy number variant (CNV)		Shared risk factors between SCZ and epilepsy: electrophysiological endophenotypes identified in a genetic deletion model of SCZ, a CNV conferring high susceptibility for SCZ and epilepsy [41, 42]
*Brain abnormalities*	- structural abnormalities within the PFC (trait marker of bipolar risk)	BD	The thickness of the cortex in the left pars opercularis was significantly lower (thinner cortex) in subjects at high-risk for BD compared to subject not at high-risk for the disease, although no differences in thickness were detected in other cortical or subcortical regions [43]
	- a respective loss in HPC area, PFC and TL volumes - reduction in grey and white matter - progressive reduction of cortical thickness - higher functional activity during cognitive tasks	Psychotic disorders	The loss in HPC/PFC/TL volumes is associated with conversion to psychosis in young subjects with prodromal symptoms of psychotic disorders, and may explain cognitive impairments reported at the early stages of the disorders. Higher functional activity during cognitive tasks may reflect a limited “cognitive reserve” [44, 45, 46]
	- microglia	SCZ	Microglia associated with at-risk symptom severity in subject at ultra-high-risk for schizophrenia, the latter being reported to have 30% chance of developing the illness within two years [47, 48]
	- thinner VMPFC	SCZ, BD	Structural MRI scans and brain connectomics revealed a correlation between polygenic risk for BD and the strength of the VMPFC functional hub within the DMN [49]
	- whole-brain neuroanatomical abnormalities patterns	SCZ	High accuracy (around 90%) prediction - using machine learning and neuroimaging data - of which at-risk individuals for psychosis will convert or not to SCZ (disease onset), thus undergo disease transition, by assessing whole-brain neuroanatomical abnormalities patterns [50]
	- decreased gray matter volumes in areas constituting hub nodes of the salience network	Psychiatric disorders	A common substrate for major psychiatric disorders has recently been defined using MRI voxel-based morphometry studies, revealing decreased gray matter volumes in the dorsal anterior cingulate cortex and the left and right anterior insular cortex. Researchers found that the common substrate was modulated by shared genetic variants; however the latter do not translate directly into higher risk for the disease [51]
*Alterations of the E-I balance*	- dysregulation of GABAergic interneurons	SCZ	A dysregulation of GABAergic interneurons at the origin of a desynchronization of cortical and cortical-subcortical circuits, of impairment of oscillatory activities and gamma-band synchronization, and of altered cognition and mood has been found [52]
	- Impaired GABA-glutamatergic coupling in the PFC	SCZ	Impaired GABA-glutamatergic coupling in the PFC alters back subcortical circuits and dopaminergic transmission, leading to the emergence of psychosis [53, 54]
	- reduction of frontocortical GABA-glutamatergic networks	SCZ	Reduction of frontocortical GABA-glutamatergic networks in young subjects at clinical-high-risk for SCZ may be involved in the worsening of cognitive inflexibility, of their sensitivity to stress, and vulnerability to drugs such as cannabis, which has been shown to interfere with GABAergic transmission [55]
*Environmental risk factors*	- Obstetric complications - Personal psychiatric background - Low IQ (<85)	Psychotic disorders	Obstetric complications, personal psychiatric background and low IQ (<85) defined as premorbid factors - comparing late (adult)-onset versus early-onset psychotic disorders - which can help predict the early onset of psychosis and therefore work towards the prevention of this early transition to psychosis in young age (children and adolescents) individuals [56]
	- Temperament - Early life adversity - Deregulation of the midbrain dopamine transmission	Psychiatric disorders	Three-factor model of early onset common to psychiatric disorders: temperament (i.e., adolescent externalizing trait), early life (childhood) adversity, and deregulation of the midbrain dopamine transmission. These three features have been proposed to increase the risk for distinct and commonly comorbid early onset of psychiatric disorders which can predict a susceptibility for this disorder, with a greater predictive power when combined [57]
	- Lifestyle and health conditions	AD	Seven risk factors for AD recently published: diabetes, midlife hypertension, midlife obesity, physical inactivity, smoking, depression, and low educational attainment. One third of AD cases worldwide can be attributed in one of those seven risk factors, which often co-occur [58]
	- Drug abuse, especially of cannabis - Social isolation - Stress - Urban living conditions - Migration	Psychiatric disorders	Common environmental risk factors [52, 59]
*Cognitive abnormalities*	- Cognitive deficits	SCZ	Cognitive deficits defined as early predictors of the development of psychosis in ultra-high risk patients for SCZ [60]
		SCZ	Psychosis vulnerability recently characterized by cognitive biomarkers defined as “top-down” inhibition impairments with the study of unaffected relatives of individuals versus healthy controls [61]
*Early-life stress*	- early exposures to stressful events and environments	Stress-related disorders	Significant impact of early life stress on the brain architecture, on the response to stress from the body and on the health later in life. Early-life stress also increases the vulnerability to several stress-related disorders in life which can interact with existing risk factors and lead to particular brain diseases [62]

From an initial insult (e.g., a brain injury), risk factors may play a key role in precipitating an already impaired brain into a specific brain disease. For example, the risk of developing a psychiatric illness depends on the complex interplay between a subject’s genetic profile and a specific set of other risk factors stemming from the environment. The identification of risk factors and biomarkers thus appears key to understand brain disease emergence and progression at preclinical (presymptomatic) and prodromal (attenuated symptoms) stages of the disease and would provide clinicians with valuable information on how to treat at-risk individuals before they develop the severe and debilitating core symptoms of a brain disease. At-risk individuals start to be under increasing scrutiny, since prevention has now become a health priority worldwide. Thus, detection of at-risk individuals is one priority, for example young adults who were exposed to several risk factors for schizophrenia [3] from conception to adolescence or subjects who undergo a brain injury [63]. Based on risk factors and biomarkers, a growing number of research recently aimed at predicting, in at-risk patients, the conversion to disease onset, e.g., the conversion to psychosis [4, 64], to Alzheimer’s (AD) [15] or Parkinson’s diseases (PD) [65], or to autism spectrum disorders [66], among other brain diseases.

Distinct risk factors have been thoroughly described across brain diseases; a non-exhaustive list is provided in Table 1, including genetic and genomic risk factors, brain abnormalities, alterations of the excitatory-inhibitory (E-I) balance, environmental risk factors, cognitive abnormalities and early-life stress.

In sum, brain diseases possess multiple common risk factors which can interact with each other. Brain diseases also share a high number of comorbidities.

## 3 Common comorbidities among brain diseases

Interestingly, brain diseases share neurological, psychiatric and cognitive comorbidities [23, 24, 27]. Until now, the status of shared comorbidities remains complex and underlying mechanisms remain to be clarified [67]. I will first describe neurological and psychiatric comorbidities across related brain diseases.

### 3.1 Neurological and psychiatric comorbidities

While neurological comorbidities often consist of epileptic seizures and dementia, psychiatric comorbidities mostly consist of psychotic episodes, depression and anxiety [68]. Psychiatric comorbidities are common in people with (all forms of) epilepsy [69], AD [70] or PD [65], as well as with autism [66], to only cite a few. Neurological comorbidities are also common in these diseases, especially epileptic seizures [71] and dementia [72]. Subjects with epilepsy present a higher risk of developing psychoses in their lives than the general population (∼10–30 % of epileptic patients may develop psychotic symptoms), and people with schizophrenia are more prone to develop (all forms of) epilepsy [69, 73]. Schizophrenia-like psychosis is one of the most severe comorbidity in epilepsy [74]. Epilepsy and schizophrenia thus share neurological and psychiatric comorbidities [74]. Epilepsy-associated comorbidities also include depression and anxiety [69]. People with AD experience epileptic seizures much more often than people without AD [71], especially in the early stages of the illness. Seizures have also been reported to occur much more frequently in at-risk subjects for AD [72]. People with epilepsy, especially with a late-onset of the disease, have been reported to present a threefold risk of developing dementia compared with the general population, which supports the idea of an epileptic AD prodrome [72]. AD thus constitutes a risk factor for epileptic seizures. A significant amount of these epileptic patients were thus at high risk for dementia [72, 75]. Such a high risk could constitute a continuum between (all forms of) epilepsy and the cognitive decline characterizing dementia. Altered brain networks at the origin of both epileptic seizures and cognitive decline may be the common denominator (shared mechanisms) between epilepsy and AD. Psychosis, depression and sleep disorders are also common comorbidities in AD [70]. In PD, patients can suffer as much from depression as from their core motor deficits [27]. In autism, autistic traits have been reported in adults with epilepsy, related to their seizure activity, with a prevalence of autism spectrum disorders in epilepsy ranging from 15% to 47% [76].

Psychiatric comorbidities have been the most studied shared feature between epilepsy and associated disorders [68]. A relatively large gap between epilepsy onset and the onset of psychiatric comorbidities suggests that “damage to key structures is necessary and that it builds up over time” [77]. Psychiatric symptoms such as hallucinations in schizophrenia may likewise arise after the build-up of compensatory mechanisms [78], e.g., over-activation of nearby sensory pathways [79], at the origin of the hallucinations.

In sum, brain diseases have in common multiple neurological and psychiatric comorbidities which may share common underlying brain network abnormalities. In the next section I will emphasize that brain diseases also share multiple cognitive comorbidities.

### 3.2 Cognitive comorbidities

A wide spectrum of cognitive impairments characterizes related brain diseases, thus are now considered a core deficit. Cognitive alterations in mental brain diseases became a major concern more than a decade ago, with the MATRICS and the CNTRICS initiatives created to improve cognition in schizophrenia [80].

In epilepsy and particularly in temporal lobe epilepsy (TLE), memory impairments and spatial disorientation are a hallmark-feature of the disease [75, 81], affecting patients' everyday life as much as their frequent epileptic seizures, if not more. The critical role of the TL in cognitive processes easily explains how imbalanced networks during TLE may lead to cognitive alterations [82], such as learning and memory deficits, social cognition deficits, among several others [83]. A vast literature highlights cognitive deficits in TLE animal models [29, 84, 85, 86] as well as in human TLE patients [87, 88]. TLE and schizophrenia also share various cognitive comorbidities [89, 90].

In AD, spatial memory is one major symptom of the disease [91], which is also affected in TLE and schizophrenia [92]. Social memory in human TLE, schizophrenia and autistic patients is impaired as well [93, 94], as is working memory [90]. Bipolar disorders are also characterized by multiple cognitive impairments [95]; the same applies for PD [28] and autism spectrum disorders [96].

In sum, it is now clearly evidenced and well-admitted that brain diseases, in addition to sharing risk factors, also share numerous comorbidities which constitute related disorders and probably associated mechanisms. I will now describe shared deficits ahead of disease onset, which are a key focus of this review manuscript.

## 4 Common alterations ahead of disease onset

While brain diseases are characterized by multiple common comorbidities after disease onset, as described above, they also share numerous deficits ahead of their onset which could be investigated in order to prevent the transition (conversion) towards disease onset. Among the shared comorbidities, it remains difficult to know which underlying mechanisms are common and how to separate contributing factors. In epilepsy for example, recent years were characterized by a paradigm shift in the association between epilepsy and its comorbidities with the identification of a bidirectional relationship between them [67, 97], in the sense that both the comorbidities associated with the disease and the disease itself can influence each other. In the case of epilepsy, contributing factors are cognitive impairments, epileptic seizures, and the side effects of anti-epileptic drugs, all of which reinforcing network remodeling. Consequently, studying shared deficits before disease onset presents a considerable advantage, as fewer confounding factors will have emerged which therefore may not have yet influenced deficits, at least not as much, especially cognitive ones. I will start to describe common neurological and psychiatric comorbidities occurring before disease onset.

### 4.1 Neurological and psychiatric comorbidities

In this section, I refer to the definition of neurological and psychiatric comorbidities which have been provided earlier in section 3.1.

In psychotic disorders, a new field has recently developed with the emergence of the clinical high-risk research whose goal is to highlight the development of major psychotic symptoms and to alter their course [4, 5]. The trajectory towards disease onset in individuals with a vulnerability towards psychotic diseases could be targeted (modulated) to delay or suppress disease onset. Using individual participant data meta-analysis with the largest samples (1676 individuals) of clinical high-risk subjects for psychosis ever collected to date with the goal of providing individualized predictions of the transition to psychosis, study showed that the internally validated prognostic performance of the model used by researchers was slightly higher than chance [4]. In large studies, the model can properly discriminate clinically-high-risk individuals with a high risk of developing psychosis from clinically-high-risk individuals with a lower risk of developing psychosis [4]. In AD, neuropsychiatric symptoms such as sleep disorder and irritability have recently been quantified as an early emerging feature of preclinical AD and predict metabolic dysfunctions (Ng et al. 2017). Similarly, in PD, REM sleep behavior disorders, anxiety and depression characterize the prodromal phase of PD [98]. I will now review early cognitive deficits across related brain diseases which occur before disease onset, a key focus of this manuscript.

### 4.2 Focus on early cognitive deficits

**Figure 1:**
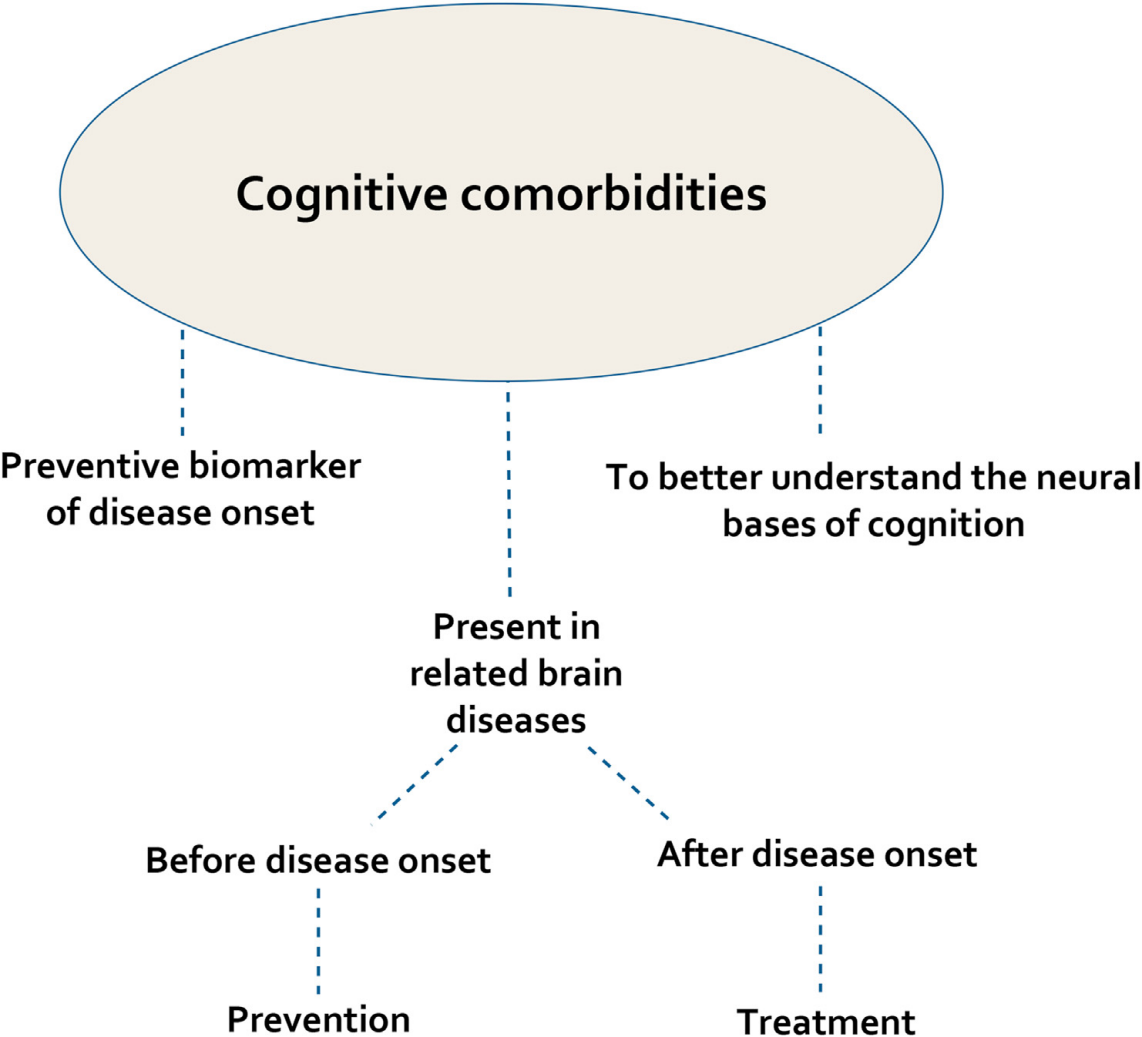
Schematics summarizing the advantage of studying cognitive comorbidities across related brain diseases. Cognitive comorbidities are present across brain diseases; they also occur both before and after disease onset. They can therefore constitute a preventive biomarker of disease onset. They can also be studied to better understand the neural bases of cognition. After disease onset, cognitive comorbidities are targeted to study appropriate treatments. Before disease onset, cognitive comorbidities can be investigated towards disease prevention since cognitive deficits may arise the earliest before disease onset, translating underlying brain network abnormalities.

While cognitive impairments are prominent after disease onset, they also present the crucial feature of occurring before disease onset (Figure 1). I will focus on their early occurrence in the remaining parts of this review and discuss the benefits of targeting such an early occurrence. While some underlying mechanisms of cognitive alteration can be modified or compensated, others cannot be reversed [12]; hence prevention appears as a valuable and cost-effective pathway to embrace [99, 100]. I will start by presenting evidence from animal studies that brain diseases share multiple cognitive deficits ahead of disease onset.

#### 4.2.1 Evidence from animal studies

##### 4.2.1.1 Epilepsy

We have shown that spatial memory deficits arise at the early stages of epileptogenesis in a TLE animal model, remain persistent after TLE onset and are correlated with a deficit in theta activity, thus constitute an early biomarker of TLE onset [29]. In this same model, preliminary results showed that TL network dynamics during REM sleep (theta coherence) was dramatically impaired before disease onset [82], suggesting that the TL network is already remodeled (altered) enough to give rise to cognitive deficits but not yet to spontaneous seizures, assuming that the TL network underlies cognitive deficits and epileptic seizures (with their focus in the TL; after disease onset) using the same resources. Models conferring a high-risk of developing epilepsy are also characterized by impaired spatial memory [101]. Five models of post-injury epilepsy were also characterized by deficits in spatial memory in rats which will further develop spontaneous and recurrent epileptic seizures [102].

##### 4.2.1.2 Schizophrenia

Spatial memory deficits have also been identified in a deletion mouse model conferring high risk of developing schizophrenia [101]. Researchers also found, using an established neurodevelopmental animal model of schizophrenia, that cognitive training during adolescence prevented the occurrence of cognitive impairments in adulthood despite a persistent brain lesion into adulthood [103]. Researchers hypothesized that such an early intervention may normalize brain function, increasing the synchrony of oscillatory activity between the hippocampi during cognitive processes, thus strengthen cognitive performance. This study supports the hypothesis whereby normalization of brain functions underlying the emergence of cognitive deficits at an early stage is beneficial towards brain disease prevention. Cognitive therapy during adolescence may therefore benefit individuals at risk for schizophrenia, rendering adolescence a critical window of intervention against disease onset [103]. So far, cognitive therapy in schizophrenia has been reported during adulthood after disease onset, namely after the occurrence of psychotic symptoms, which may explain the high rate of failure of such a therapy compared to intervention in younger subjects [104]. The advantage of this early time window of intervention is that the deficits do not yet comprise the full spectrum of (positive, negative and cognitive) core symptoms of the disease. Adolescence could be considered a critical time window when cognitive deficits could be targeted and potentially modify the trajectory of the disease towards its onset. Interestingly, restoring synchrony deficits was sufficient to increase cognitive performance in the same animal model [105].

##### 4.2.1.3 Alzheimer’s disease

Spatial memory is the earliest symptom precursor of the disease in several animal models , both in rats and mice [106, 107]. Cognitive deficits follow the same pattern of evolution in human-mimicking APP mouse models as in humans [108]. Starting with alteration of spatial working memory, disease progression is associated with deficits in associative learning and reference memory, followed by impairments in recognition memory later on [108]. Similarly to genetic deletion models conferring high risk of developing epilepsy and schizophrenia [101], risk factor animal model of AD has also been characterized by memory deficits [109].

In summary, recent research on animal studies showed that brain disease do share cognitive deficits before disease onset. I will now report evidence in human subjects.

#### 4.2.2 Evidence from human studies

As for animal models, spatial memory is the earliest symptom precursor of AD [91] and has been shown to be impaired as well in people with TLE or schizophrenia [110, 111]. Social memory and cognition in epilepsy and schizophrenia patients are also impaired [89, 94], especially working memory [90].

##### 4.2.2.1 Schizophrenia

Cognitive deficits have now been proposed to occur as primary symptoms of the disease [112, 113] while well-known positive and negative symptoms have been suggested to rather manifest as secondary, hypothesized to occur as an adaptive response, namely, to compensate for the aberrant network dynamics. Already in the 90s, cognitive and motor impairments occurring before the psychotic symptoms which mark disease onset were reported in children [114]; the same applies to young adults who later developed schizophrenia [115] and to children at genetically high-risk for schizophrenia [116]. Impaired social cognition in individuals with schizophrenia is a key factor in predicting conversion to full psychosis in at high-risk but asymptomatic subjects [27]. In the 22q11.2 deletion syndrome model of schizophrenia (patients with such genetic deletion have an elevated risk of ∼30% of developing schizophrenia), early cognitive decline is a robust biomarker of the risk of developing a psychotic illness [117]. In individuals at ultra-high-risk (UHR) for schizophrenia, cognitive deficits arise ahead of the onset of psychosis, with cognition being modulated by polygenic risk scores for resilience [118]. The genetic profile of subjects at UHR for schizophrenia appears to modulate the cognition in individuals who do not convert to psychosis during the one-year follow-up. Cognitive impairments can thus be predictive of the conversion of at-risk individuals to psychosis, with the intervention of genetic risk factors playing an important role in the conversion.

##### 4.2.2.2 Epilepsy

Study showed that over time (25-year longitudinal study), subjects who had developed late-onset epilepsy had a faster cognitive decline compared to the subjects who did not develop the disease [119]. Cognitive decline ahead of late-onset epilepsy therefore appears informative of disease development. In new onset epilepsies, 70% of newly diagnosed patients already presented cognitive impairments at the onset of epilepsy (Witt and Helmstaedter 2015). Importantly, around 50% of late-onset epileptic patients have been diagnosed with mild cognitive impairments (MCI) at seizure onset before taking any treatment against seizures, which means that these deficits were not due to the side effects of antiepileptic drugs.

##### 4.2.2.3 Alzheimer’s disease

Cognitive deficits already occur during the preclinical (asymptomatic) phase of the disease with impairments in episodic and semantic memory, in executive function and attention, in visuospatial memory and in verbal recall as the disease progresses towards the phase of MCI, both phases occurring ahead of AD onset [108]. As the progression transitions into the dementia stage of AD, all cognitive domains become affected [108]. In young adults at genetic risk for AD, spatial memory deficits were observed [91], suggesting that decades before a potential disease onset, cognitive deficits have already emerged [120]. Chin & Scharfman (2013) however reported that cognitively intact subjects at genetic risk for AD displayed similar cognitive performance for multiple tasks as subjects not at-risk for AD, which suggests that at this time, network abnormalities may not have emerged yet into cognitive deficits; a reason for this could be that genetic risk for AD is slower in precipitating into AD than the risk of having undergone an initial brain insult, for example (cf. section 4.4). Pressure for early intervention in order to prevent the progression of dementia and initiate treatment administration in patients with MCI is one major health priorities. However so far, clinical trials have not provided yet very promising results [121].

##### 4.2.2.4 Parkinson’s disease

Similar to epilepsy, schizophrenia, and AD, cognitive impairments are common in PD and can arise at an early stage of the disease, when cognitive deficits are still asymptomatic [122]. Predictive biomarkers, such as MCI occurring early in PD stage, have become under crucial focus to slow down or prevent PD dementia and may offer prognostic information [28]. MCI exist in both PD and AD and constitute in PD an intermediate step between normal cognition and dementia [28]. Using a cohort of 7386 individuals, the Rotterdam Study associated poor global cognition with a higher risk of developing the disease [123], suggesting cognitive impairment as a prodromal PD feature [124]. Studies confirmed cognitive impairments in at-risk individuals for PD but not yet diagnosed with the disease [125, 126], with converters to PD onset having lower cognitive performance on various cognitive domains, especially in the field of executive function, working memory, and global cognition, than non-converters [127]. Cognitive dysfunction could thus predict conversion to PD [126].

Altogether, these results show that cognitive alterations precede the onset of related brain diseases and that ongoing research goes more and more in the direction of identifying cognitive deficits as primary symptoms of brain diseases before the latter progress and specify with the emergence of core symptoms which bidirectionally influence each other and become confounding factors.

### 4.3 Associated network abnormalities

It has been shown that what all shared comorbidities between related brain diseases have in common are network dynamics abnormalities [23]. In this section, I will report network abnormalities underlying early cognitive deficits ahead of disease onset. First, I will describe two common network abnormalities associated with early cognitive deficits. I will then report abnormal brain correlates per brain disease.

#### 4.3.1 Theta rhythm

Oscillatory abnormalities are often described as an underlying feature of the pre-onset period in several related brain diseases. A common substrate of brain diseases is altered theta rhythm underlying cognitive deficits. While we found no alterations of gamma activity in the early stages of TLE in a rat model, we identified a significant and persistent decrease of theta power and frequency during epileptogenesis which correlated with spatial memory deficits [29]. Theta coherence between brain regions of the TL was dramatically altered during REM sleep, a theta-dependent behavior, in TLE rats before disease onset (personal communication). Altered theta activity was also described in five models of post-injury epilepsy (mice and rats) [102], in a 15q13.3 microdeletion model associated with high risk of developing schizophrenia and epilepsy [101], in schizophrenia [128], especially between the prefrontal cortex and the hippocampus [129], in human newly diagnosed TLE [87], and in a risk factor model of AD [109]. Impaired theta dynamics were also reported in human subjects with TLE associated with working memory deficits compared to subjects with TLE depicting no such deficits [88]. Theta dynamics thus appear as a general biomarker for epileptogenesis in animal models of TLE and as a predictor of cognitive deficits in subjects with TLE, with greater decrease in theta correlating with earlier TLE onset in several animal models of post-injury epilepsy [102].

#### 4.3.2 Gamma rhythm

Altered gamma activity is another common substrate of brain diseases, especially highlighted in schizophrenia, and associated with cognitive deficits, particularly spatial working memory deficits [130]. This is not surprising since gamma cycles are nested into theta cycles towards input potentiation, encoding and information processing, especially during working memory [82]. In a murine model of AD, a significant decrease in behaviorally-driven gamma oscillations before the occurrence of plaque formation and cognitive decline was reported [131]. Using optogenetics to drive fast-spiking parvalbumin interneurons at gamma frequency induced a decrease of specific amyloid-β isoforms [131].

#### 4.3.3 Schizophrenia

Researchers showed decreased prefrontal cortex – thalamic functional connectivity (executive control network) in the early stages of psychosis which correlates with somatomotor-thalamic hyperconnectivity in patients, suggesting a shared pathophysiological mechanism [132]. A correlation between thalamic-frontoparietal connectivity and cognitive performance was obtained. The mediodorsal thalamus has indeed been recently highlighted as being a crucial partner of the prefrontal cortex in working memory processing [128], with working memory widely impaired in schizophrenia [133]. Thalamic dysconnectivity has been associated with conversion to psychosis in subjects at clinical high-risk for psychotic disorders and correlated with symptom severity, suggesting that thalamic connectivity may support prognostic (risk of conversion) towards disease onset [134]. Studies also found an impairment of the fronto-temporal network in UHR subjects for developing psychosis [135], with prefrontal hyperconnectivity reported to be predictive of symptoms [134], especially its ventrolateral part [136]. Study showed that prodromal subjects differed from controls in functional connectivity pattern of the inferior frontal gyri (Broca’s area) [137], with risk of psychosis characterized by a decreased engagement of prefrontal regions involved in cognitive control [138].

#### 4.3.4 Alzheimer’s disease

While the assumed hypothesis stated that the development of histopathological abnormalities preceded the aberrant dynamics underlying cognitive comorbidities, more and more evidence showed that abnormalities within TL dynamics are the primary marker of the disease. Studies showed that aberrant synchrony within TL dynamics - e.g., abnormal theta-gamma coupling in the subiculum of AD transgenic mice or impaired *in vivo* gamma oscillations in the medial entorhinal cortex of knock-in mouse model - was a primary emerging feature related to AD [139, 140]. Initial impairments of hippocampal and entorhinal network activity before the accumulation of amyloid-beta (Aβ) thus represent an early biomarker of AD [91]. In a mouse model of tau and Aβ aggregation, tau accumulation in an already impaired parieto-hippocampal network underlying early spatial memory deficits was reported at the early stage of AD [141]. Oscillatory changes were also described in the hyperglycemia risk factor animal model of AD, reflecting hippocampal and prefrontal network dynamics abnormalities and associated with spatial working memory deficits [109]. Interestingly, Chin & Scharfman (2013) reported that subjects at genetic risk for AD displayed an hyperactivation of their medial TL but no differences in cognitive performance compared to subjects not at-risk for the disease; these results suggest that this hyperactivation in presymptomatic/prodromal stage of the disease reflects compensatory mechanisms [142]; nevertheless, with the evolution of the disease, the persistent hyperactivation “precipitates cognitive decline and, perhaps, psychiatric symptoms such as depression that are also associated with hippocampal hyperactivity.” [81]. These results highlight a window of opportunity where the hippocampus could be targeted in order to prevent cognitive decline and psychiatric comorbidities to arise ahead of AD onset.

#### 4.3.5 Parkinson’s disease

Patients characterized by MCI displayed a decrease in the volumes of their medial TL as well as modifications on PET (Positron Emission Tomography) imaging which contrast with subjects characterized by no MCI [28]. The identification of abnormal brain correlates associated with early cognitive deficits which have been reported during the prodromal stage of PD in at-risk subjects who already present early signs of the disease is clearly missing.

#### 4.3.6 Epilepsy

Despite the lack of specific TLE structural abnormalities, patients newly diagnosed with non-lesional TLE displayed few cognitive deficits and EEG abnormalities such as focal epileptiform discharges and altered theta rhythm [87]. These results suggest that in non-lesional TLE, early cognitive impairments are related to an underlying pathology involving brain networks beyond the TL epileptic focus rather than to TLE-associated factors. Study also showed that the seizure-free period preceding TLE onset is characterized by lower inter-hemispheric resting-state functional connectivity at fronto-temporal regions and by modifications in low-frequency oscillations [143], highlighting that widespread altered intrinsic functional connectivity could serve as an early diagnosis in at-risk subjects for epilepsy.

Altogether, these results highlight dysfunctional brain networks as a transdiagnostic pathophysiological mechanism leading to the emergence of early cognitive deficits before disease onset. These network abnormalities may arise as a common ground for brain diseases, emerge as early cognitive deficits and progress into a specific brain disease condition. Endophenotypes characterizing a pathological brain may arise before risk factors come into play and precipitate altered functional brain networks into particular disorders and a specific brain illness. I will now describe, in the last section of this review, a proposed scenario related to brain disease etiology and discuss how early cognitive deficits could be targeted towards brain disease prevention.

### 4.4 Proposed scenario on brain diseases etiology: a potential common cause

**Figure 2:**
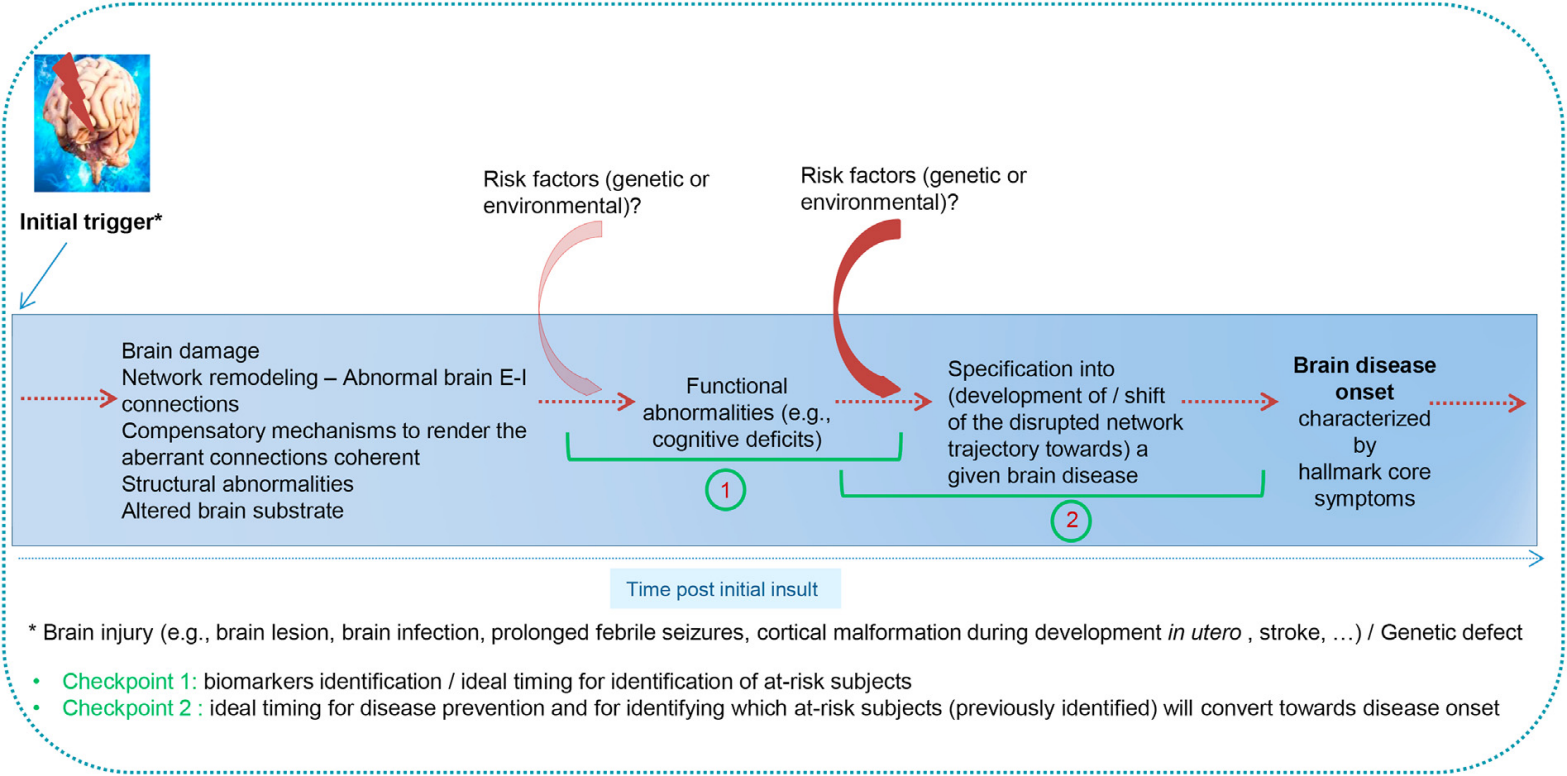
Proposed scenario on how brain disease develops from an initial insult (trigger). An initial insult may occur as a consequence of brain damage (e.g., a structural abnormality such as focal cortical dysplasia, or a brain injury after prolonged febrile seizures), malformation (brain abnormality during development such as lissencephaly or polymicrogyria) or dysfunction (genetic abnormality, for example of an ionic transporter). A brain injury leads to a brain abnormality which gives rise to altered excitatory-inhibitory (E-I) connections which the brain tries to render coherent via compensatory mechanisms. These abnormal connections may cause network remodeling characterized by impaired network dynamics which are at the origin of abnormal functioning emerging as early cognitive impairments. The initial insult can also occur as a consequence of a genetic defect causing an altered brain substrate. The latter may impair E-I neuronal transmission which similarly will lead to brain dynamics abnormalities and impaired brain functions such as cognitive deficits. For example, a genetic mutation for a specific ion channel, critical to insure neuronal transmission, could lead to deleterious E-I transmission and altered brain functioning. The initial insult renders the brain more prone to develop a brain disease which may manifest as a consequence of the progression of network reorganization. This phenomenon occurs ahead of any specification yet for a brain disease, as a common cause of an at-risk brain for related brain diseases. Genetic susceptibility for a particular disorder or trait (e.g., stress) could also be at play. Genetic (e.g., risk genes) and/or environmental (e.g., stress, urban rearing) risk factors may intervene later and precipitate the trajectory of a yet disrupted network towards the specification of a particular brain disease such as epilepsy, schizophrenia, or autism. The figure indicates checkpoints on when to act - according to the time post-initial insult - in order to (i) detect at-risk subjects, (ii) identify biomarkers, and (iii) predict whether at-risk individuals will convert towards disease onset, in order to serve brain disease prevention. Early cognitive impairments might be the first phenotypic (clinical/functional) expression of an underlying disrupted brain network translating an initial brain insult and the development of abnormal brain connections and associated mechanisms. In order to detect, among at-risk individuals, which subjects may start developing a given brain disease, it is important to be able to discriminate brain network dynamics abnormalities which are still reversible from abnormalities embedded towards a progressive and increasingly irreversible decline of brain damage and brain functioning. This decline may be enrolled with maladaptive changes and disease propagating (precipitating) factors.

An initial insult (a brain injury) occurs – e.g., cortical malformation during development, a stroke, or a traumatic brain injury – and induces structural brain abnormalities or miswiring between neuronal connections over time (Figure 2) [63, 144], given the fact that brain injuries constitute risk factors for most brain diseases [145, 146]. These alterations constitute the trigger of network reorganizations with the remaining (spared) E-I neurons which in the process generate aberrant connections between each other. From there, the brain progressively tries to render those aberrant connections coherent (adaptive response) with the build-up of compensatory mechanisms to re-establish brain homeostasis. The aberrant E-I connections may primarily lead to modifications in the global E-I balance, with glutamatergic and GABAergic transmissions becoming decoupled, at the origin of changes in oscillatory features – emergent properties of brain networks, modifications in the temporal coordination that results from brain rhythms to encode (potentiate) and retrieve relevant information, modifications in synaptic potentiation between neuronal ensembles as well as changes in neural synchrony within and between neural networks. Afferent multifaceted and multimodal input activity patterns may thus be wrongly processed as relevant or irrelevant (potentiated or depotentiated), leading to altered behavior and cognitive deficits [82]. I hypothesize that cognitive deficits emerge as a primary expression (phenotype) of network reorganization associated with an alteration of the E-I balance, thus of aberrant neural synchrony which influences the way brain networks process and integrate a message to form memories and drive choices and actions. Cognitive deficits emerging early as the clinical expression of network abnormalities could constitute a etiopathophysiological mechanistic ground for brain diseases, at least for those emerging from an initial brain insult leading to network reorganization. Cognitive deficits may thus appear as a primary condition preceding the specification of brain networks towards a given brain disease. As synchronization of neuronal responses is critical for information processing, allowing the communication between local and remote brain areas necessary for brain computations and cognitive processing, it may be at the origin of related brain diseases characterized by cognitive deficits [147]. Inter-individual differences shaping behavior and giving rise to vulnerability trait, as well as genetic and environmental risk factors, may precipitate brain networks expressing early cognitive deficits towards the development of a specific brain disease. Shared cognitive impairments before disease onset may thus constitute convenient biomarkers in at-risk subjects who suffered a brain injury. Within the course of brain diseases, whether cognitive alterations characterize a brain already embedded towards (developing) a particular brain disease or whether they simply translate an at-risk brain for developing potential related brain diseases before risk factors and/or adverse conditions come into play and precipitate the brain towards a specific brain disease condition may depend on their time of occurrence post-injury [63, 102].

## 5 Conclusion and future directions

Brain diseases have a lot in common, including risk factors and comorbidities, especially cognitive deficits before their onset. Identifying underlying pathophysiological neuronal correlates of early cognitive deficits common to related brain diseases in animal models towards the prevention of disease onset is the next step to embrace and the current research interest of my group. Targeting network dynamics abnormalities underlying early cognitive alterations may, on one hand, help understand the development (network dynamics trajectory) towards disease onset therefore help modify the course of the disease. On the other hand, it may help follow-up at-risk subjects for a given brain disease and predict whether these subjects will develop or not such a disease; in that case, therapy interventions may be of help to (delay or) prevent the transition (modify the trajectory) towards disease onset in these individuals. Restoring network dynamics with non-invasive techniques such as neurofeedback may rescue cognitive performance and delay or prevent disease onset. Fronto-temporal networks and their underlying theta and gamma dynamics constitute a promising target for preventive intervention therapies involving the restoration of network dynamics abnormalities and rhythmopathies associated with early cognitive deficits. The identification of electrophysiological endophenotypes will present the advantage of associating activity patterns and network dynamics with behavior and cognition since the latter (behavioral and cognitive states) modulates the former (field and neuronal activity).

Towards prevention, three major steps are 1) to identify the neuronal correlates associated with early deficits across related brain diseases, 2) to predict whether at-risk subjects will develop the disease by targeting these neuro-correlates, and 3) to prevent disease onset by modulating these neuro-correlates and hopefully modify the trajectory towards disease onset to delay or suppress the latter.

## Declarations

### Author contribution statement

The author listed has significantly contributed to the development and the writing of this article.

### Funding statement

This work was supported by 10.13039/501100006209Radboudumc.

### Data availability statement

No data was used for the research described in the article.

### Declaration of interest’s statement

The authors declare no conflict of interest.

### Additional information

No additional information is available for this paper.
